# Educating smokers about the risk of blindness – insights to improve tobacco product health warning labels

**DOI:** 10.1186/s12971-016-0094-7

**Published:** 2016-08-19

**Authors:** Ryan David Kennedy, David Hammond, Marlee M. Spafford, Ornell Douglas, Julie Brûlé, Geoffrey T. Fong, Annette S. H. Schultz

**Affiliations:** 1 Propel Centre for Population Health Impact, University of Waterloo, Waterloo, ON N2L 3G1 Canada; 2 Institute for Global Tobacco Control, Department of Health, Behavior & Society, Johns Hopkins Bloomberg School of Public Health, Baltimore, MD 21205 USA; 3 School of Public Health and Health Systems, University of Waterloo, Waterloo, ON N2L 3G1 Canada; 4 Faculty of Science, University of Waterloo, Waterloo, ON N2L 3G1 Canada; 5 École d’optométrie, Université de Montréal, Montréal, QC H3T 1P1 Canada; 6 Department of Psychology, University of Waterloo, Waterloo, ON N2L 3G1 Canada; 7 Ontario Institute for Cancer Research, Toronto, Ontario M5G 0A3 Canada; 8 College of Nursing, Rady Faculty of Health Sciences, University of Manitoba, Winnipeg, MB R3T 2N2 Canada

## Abstract

**Background:**

Health warning labels (HWL) on tobacco products help educate smokers about the health effects from smoking; however, there is a need to improve HWL content including images and text to increase effectiveness. In Canada, a HWL was created that communicates smoking’s causal association with “blindness” from age-related macular degeneration (AMD). This study surveyed Canadian optometrists about their opinions regarding the image and text used in the “blindness” HWL.

**Methods:**

An online survey was sent to all 4528 registered Canadian optometrists. Respondents were asked if the HWL conveyed important and believable information, and if the picture was appropriate. Optometrists were invited to make open-ended comments about the label which were analyzed using a qualitative analysis framework suitable for health policy evaluation. Frequency distributions were calculated for closed-ended questions.

**Results:**

The survey was completed by 850 respondents (19 %). Most respondents (90 %) reported the message was believable/somewhat believable; while 35 % felt the picture was “too graphic”. Some respondents reported in their open-ended comments that they were concerned the HWL was internally inconsistent because it reports there is “no effective treatment in most cases” for AMD but the image depicts someone undergoing surgery. There was concern that this may discourage patients from seeking needed treatment.

**Conclusion:**

The majority of Canadian optometrist respondents were in agreement that the new, “RISK OF BLINDNESS” pictorial HWL includes important, believable information. Some optometrists had concerns that the HWL included a confusing message or a message that may discourage some patients from pursuing treatment for AMD. Future development of blindness-related HWL should seek practitioner input.

## Background

**Fig. 1:**
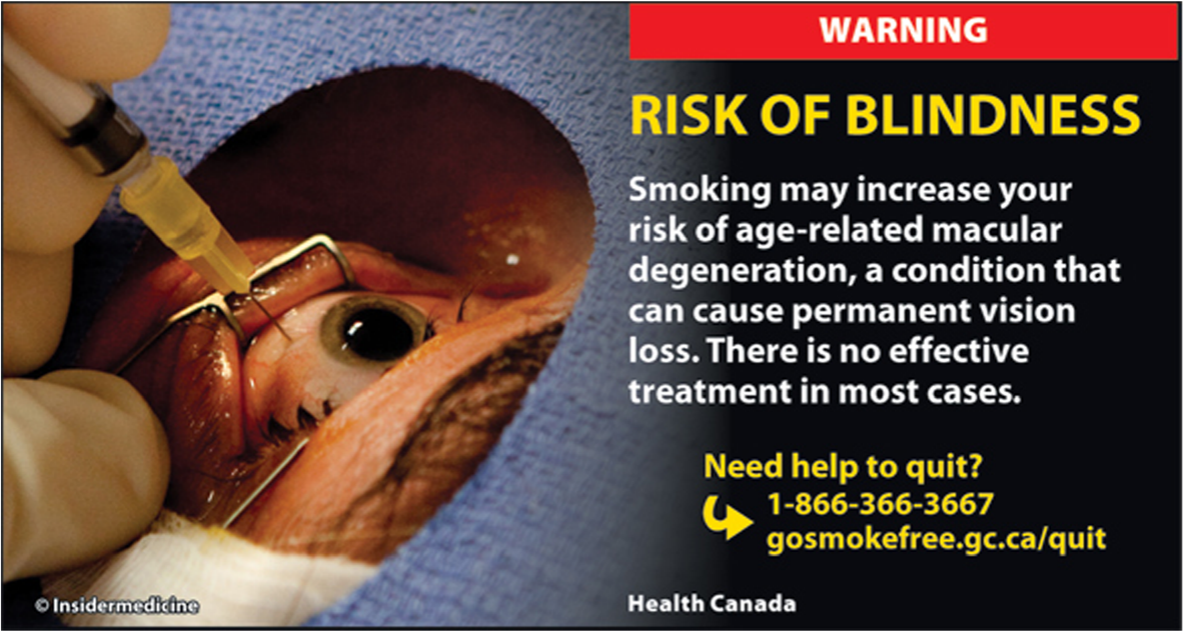
Canadian Health Warning Label. Copyright © 2012. Insidermedicine [26]

Health warning labels (HWL) on tobacco products provide an effective way to educate smokers and non-smokers about the harms of tobacco use [1, 2]. The use of images with pictures is more effective than text only warnings [2]. A recent State of the Evidence report on HWL highlighted the need for more research to inform optimal HWL message and image content in particular to understand potential interactions between various design elements [1]. In March 2012, Health Canada introduced 16 new pictorial health warning labels for cigarettes [3] including, for the first time, a label with the message “WARNING - RISK OF BLINDNESS” (see Fig. 1). The text in this HWL describes the causal association between smoking and age-related macular degeneration (AMD). AMD is a condition that causes central vision loss that is normally permanent and significant. When inhaled, the chemicals in cigarette smoke reduce a person’s oxygen intake, which in turn damages the body’s vascular system [4]. This can damage the tiny blood vessels in the macula at the back of the eye, leading to the development of AMD. Tobacco use is the most important, preventable risk factor for AMD [5]. Current smokers have four times the risk of developing AMD compared with non-smokers [6], and may develop AMD about 10 years earlier [7]. AMD is the leading cause of vision loss in Canada. It is estimated that AMD affects one million Canadians and that this number will double by 2031 [8].

Studies have shown that using “loss of eye sight” in Australian and New Zealand media campaigns is effective at increasing cessation seeking behavior including calling quitlines [9, 10]. Adults and youth are fearful of going “blind” and may be motivated to quit smoking if they believe that it could lead to vision loss [11]. Only a few countries, including Australia, have used pictorial HWLs that include messages about vision loss and smoking. The Australian “blindness” HWL is associated with increased smokers’ knowledge of this important health effect [12, 13], yet both smokers and non-smokers have reported difficulty understanding how vision loss is caused by smoking [14].

Prior to the Health Canada HWL, there had been no national public awareness campaigns linking smoking to vision related disorders and few Canadian adult smokers (13 %) reported that they believed there was a link between smoking and “blindness” [12]. Previous research with Canadian optometrists identified that there was support for the development of education campaigns that increase patients’ knowledge about smoking and blindness and help to legitimize optometrists’ discussions with patients to help educate them on the impact of smoking on ocular health [15].

Optometrists in Canada are at the forefront of providing vision care [16] and are well positioned to address questions that might arise from patients related to smoking and vision loss. The current study sought to assess Canadian optometrists’ opinions about the Health Canada “RISK OF BLINDNESS” HWL, including the picture used and the message content.

## Methods

### Data collection

The research team partnered with each of Canada’s 10 provincial optometry regulatory authorities and the Yukon Territory’s Department of Community Services, who sent an email to all practicing community optometrists in Canada (*N* = 4528) in March, 2012. The email explained the study and included a link to an online bilingual (English/French) survey. In some cases the email was delivered by the provincial regulatory authority and in others by the Survey Research Centre at the University of Waterloo depending on the privacy policy of the regulator. Optometrists received the initial email invitation at the end of February 2012, and if they had not completed the survey, they received weekly reminder emails until the end of March 2012. Participants could complete the survey in either of Canada’s two official languages: English and French. No incentive was provided for participation; at the end of the survey respondents could request health education materials about smoking and eye health.

### Sample

The response rate among optometrists was 19 % (850 responses). The survey was completed in English (77.1 %, *n* = 655) and French (22.9 %, *n* = 195), similar to the national proportion of English and French speakers in Canada [17]. The majority of respondents were women (59.9 %, *n* = 501), similar to Service Canada’s estimate of women comprising 64 % of the optometry labor workforce in 2012 [18]. Almost all respondents reported that they were a never-smoker (90 %), and no respondent identified as a current smoker.

### Analysis

Optometrists were shown the new “RISK OF BLINDNESS” HWL which was not yet in circulation on Canadian cigarette packages. Respondents were asked six closed-ended survey questions about their thoughts including: if the new label conveyed important information about the harms of smoking; how believable the label was; how effective the label might be; if the picture was appropriately graphic; and how the label might influence smokers’ motivation to quit and persuade non-smoking youth to not start smoking. Response options were “yes”, “somewhat yes”, “somewhat no”, and “no”. Optometrists were then asked, “Do you have any further comments regarding this label?”.

Frequencies were calculated for closed-ended questions. In the case of non-responses, the reported proportions were based on the number of respondents for each question.

Open-ended responses were analyzed by two bilingual researchers (RDK, OD) and coded using classifications identified a priori consistent with the Framework Approach [19], a method of qualitative data analysis recommended for health research. Classifications were informed by the design elements present in the health warning label. These included comments pertaining to the label’s: (1) picture and (2) textual content in terms of the message, cessation supports, and attribution to Health Canada. Representative excerpts are reported. Other emergent ideas were identified and included in the final presentation of the findings. Each quote presented is from a unique respondent.

## Results

### Close-ended responses

Almost all respondents (93.5 %, *N* = 786) agreed (‘yes’ or ‘somewhat yes’) that the new HWL “RISK OF BLINDNESS” provided important information about the harms of smoking. The majority of optometrists agreed (89.5 %, *n* = 751; ‘yes’ or ‘somewhat yes’) that the HWL included a believable message. More than three quarters of optometrists agreed that the image would be effective for the general public (78 %, *n* = 656; ‘yes’ or ‘somewhat yes’). Optometrists were asked ‘Is the image too graphic?’ Over one third (35 %, *n* = 295) responded ‘yes’ or ‘somewhat yes’. When asked if they thought the HWL would increase smokers’ motivation to quit, more than half (65.8 %, *n* = 551) replied ‘yes’ or ‘somewhat yes’. Less than half (49.8 %, *n* = 417) replied ‘yes’ or ‘somewhat yes’, that the HWL would persuade non-smoking youth not to start smoking. Table 1 provides responses to closed-ended questions regarding optometrists’ impressions of the HWL.

**Table 1:** Responses to closed-ended questions

	Yes	Somewhat yes	Somewhat no	No	Missing
Does the new warning label provide important information about the harms of smoking?	53 % (*n* = 447)	40.3 % (*n* = 339)	4.0 % (*n* = 34)	2.5 % (*n* = 21)	9
Is the message believable?	44.2 % (*n* = 371)	45.3 % (*n* = 380)	8.0 % (*n* = 67)	2.5 % (*n* = 21)	11
Is the image effective for the general public?	38.8 % (*n* = 326)	39.3 % (*n* = 330)	16.2 % (*n* = 136)	5.7 % (*n* = 48)	10
Is the image too graphic?	9.1 % (*n* = 76)	26.1 % (*n* = 219)	22.4 % (*n* = 188)	42.5 % (*n* = 357)	10
Do you think the warning label would increase smokers’ motivation to quit?	12.2 % (*n* = 102)	53.6 % (*n* = 449)	22.8 % (*n* = 191)	11.5 % (*n* = 96)	12
Do you think the warning label will persuade non-smoking youth not to start smoking?	8.8 % (*n* = 74)	40.9 % (*n* = 343)	29.1 % (*n* = 244)	21.1 % (*n* = 177)	12

### Open-ended responses

Approximately 27 % of survey participants provided open-ended comments about the new HWL (*n* = 233). There were no comments about the HWL’s Quitline contact options or the label’s author (Health Canada). Comments about the image and text were classified by the research team as positive or negative. Table 2 includes representative ‘positive’ and ‘negative’ quotes that address the HWL’s image and text, and suggestions for changes to the image and text. In addition, select quotes are included below that provide more general comments of the HWL.

**Table 2:** Canadian optometrists’ comments on the pictorial health warning label ‘RISK OF BLINDNESS’

Picture - Positive comments	Text - Positive comments
« I find it quite striking! » [translated from French]	I think the headline is quite clear, which is good. People often don’t read the entire thing.
«For some, vision is the worst thing you could lose… For others, touching the surface of the eye, particularly with a needle would be truly unpleasant, … So with these 2 points of view, it is a good [warning] label» [translated from French]	… Canadians highly value vision and fear blindness. This may be more effective labeling than previous ones highlighting other diseases and risk of mortality.
It may be graphic, but it needs to be.	“Warning message is clear”
Now that’s graphic! If this doesn’t make someone **** their pants over smoking, nothing will.	People have more fear of losing their vision than of lung cancer. So I think that this type of [message] can influence smokers to think about quitting
Picture - Negative comments	Text - Negative comments
Image is too graphic. Public will be too overwhelmed and will simply disregard the image and the information.	I don’t like this message. There are many patients that suffer from wet macular degeneration that are non-smokers and have been their entire lives.
« The needle is not really necessary, a little too shocking. Why so much sensationalism to get a message across… » [translated from French]	« It can discourage and scare people affected by AMD [from seeking treatment] even if they do not smoke. » [translated from French]
«At a time when there are disgusting images on cigarette pacts, the impact of this photo… seems less shocking. » [translated from French]	«In my opinion… the text is a little long. » [translated from French]
«The photo doesn’t show a problem with vision or eye health, only surgery which doesn’t demonstrate the seriousness of the situation in my opinion, and few people will read the description beside… We need something more dramatic. » [translated from French]	I feel that it would improve its effectiveness if there was a statistic to reinforce the increased risk of macular degeneration and severe vision loss.
Picture is too technical, too abstract - difficult to relate to.	It lacks the message that the disease will affect [patients] at a younger age
Picture - Suggestions	Text - Suggestions
I believe an eye with a thick cataract visible might be more effective since it actually visually shows the diseased eye.	I hate the word ‘may’. Also, vision loss doesn’t sound as blunt or scary as ‘blindness’ would. The [HWL] should read: Smoking increases your risk of.....permanent blindness.
Maybe using a picture of what a person with macular degeneration sees compared to a normal person would be more effective and worrisome to the patient.	The wording needs to be more threatening, as teenagers won’t worry about an age related disease.
I feel like this patient [appears too old], with the wrinkles around the eyes. Smokers will view this as a ‘this happens to old people’ I can just quit later, before this happens to me. It should be more relatable to more demographics.	My feeling is that the Age-related part of ARMD on the label may slightly reduce the effectiveness of the label. The general public may get the impression that it’s more a factor of age instead of smoking.. I think that age-related should be dropped and just written as …risk of macular degeneration
Remove the blue cloth surrounding the eye and change the colour of the needle	[add that] age-related macular degeneration, the leading cause of blindness in Canada..
the image should zoom in another 10–20 %, cropping more from the top and right sides, and focusing more on the eye itself	…add something about not being able to drive, read or see faces of friends/family.
[the picture] needs to be more graphic	The label could indicate that retinal damage can be reduced with certain lifestyle changes such as quitting smoking.

Many respondents were enthusiastic about the HWL. One respondent felt relief that Health Canada “…*finally put a warning label about eye disease on cigarette packs*”. Respondents commented on how strategic it was to include eye disease in a warning label; “*for some, vision is the worst thing you could lose”.* Other respondents described how the new label would support their discussions with patients; “*Patients do not always want to believe us, but with such a photo, I have a fantastic argument.”* Another respondent felt that the HWL could help reinforce discussions with patients and further support quit seeking behavior:

*I believe that if a person has seen an optometrist and then warned of this danger - then when they see this label it may help remind them of what was said during their eye exam and may help them to choose to quit.*

Another respondent felt this HWL could “*help motivate patient[s] to quit smoking.”* Several respondents commented on how the HWL may influence youth not to start smoking. One respondent commented that the image was very graphic, but justified the image explaining, “*I suppose a gory picture attracts more attention especially from youth.*” Several respondents were skeptical that a HWL depicting a health outcome associated with older people would resonate deeply with youth, “*if you are looking to persuade youth to not smoke, the threat of an age-related disease is not likely going to be of much help.”*

Respondents also provided critical comments about both the image and text used in the HWL. Many respondents felt the image was too graphic or shocking. One respondent commented that the use of a needle may only serve to scare people who are afraid of needles. Others recommended the wording should be more definitive; avoid saying that smoking “may increase your risk” and instead state that smoking “does increase your risk”. Others commented that the text and image combination could be confusing to the general public because the label indicates that there are no effective treatment options for AMD, but the image on the label shows someone undergoing treatment. Respondents voiced concern that some of their patients with AMD may be discouraged by the HWL from seeking recommended treatment because treatment options are described as being “ineffective in most cases.” One respondent highlighted how the ad’s inconsistent message could impact their credibility,

*The ad does not explain what AMD is nor its effects on vision. Furthermore, the text indicates there is no effective treatment, and yet the image shows an intraocular injection. This only serves to confuse an already uneducated public, and will only decrease our credibility*.

Some respondents provided design suggestions for a different image that could depict the typical central vision loss that occurs when they have AMD*:*

[I suggest] *a simulation of how a person's vision is affected with AMD. For example, the appearance of print in a magazine with normal vision vs. vision with AMD. Or the appearance of a child's face, when viewed with normal vision vs. vision with AMD. Sticking a needle in someone's eye is gruesome, but does not describe the vision loss caused by AMD*.

Others suggested including messages about how this type of vision loss would impact quality of life, including loss of the drivers’ license or inability to text or use a smart phone.

## Discussion

The results from this online survey indicate that the majority of Canadian optometrist respondents agreed that the new ‘RISK OF BLINDNESS’ HWL includes important, believable information that can help educate smokers about this important health effect. Most optometrists agree that this HWL may be helpful to motivate smokers to quit, and approximately half agree the HWL may discourage young people from starting to smoke, although some respondents pointed out that the use of age-related health outcomes may limit the HWL’s impact on some younger smokers. Some respondents suggest the new HWL may reinforce messages optometrists give their patients about the harms of tobacco use, and the benefits of quitting. Some optometrists were very enthusiastic about the HWL, and thankful that Health Canada had included this important health effect from smoking in its new collection of tobacco product warning labels.

Some important critiques were provided about the image and text used in the blindness HWL. Some optometrists felt this label could be misleading or confusing for the general public because there was not a consistent message from the text and image regarding whether AMD is treatable. This inconsistency may dissuade or discourage people with AMD from pursuing important treatment when it is viable.

This study has many strengths including that all practicing optometrists in Canada were contacted to participate in the survey and that the survey collected both qualitative and quantitative data. The study has limitations, particularly as it pertains to policy and practice given that the respondents are limited to optometrists who do not use cigarettes. Further, the optometrists were seeing the HWL for the first time as it was not yet in circulation therefore their perspectives and thoughts about the label’s possible impact on behaviour or role in preventing youth were not informed by experiences in their practice. The HWL was also displayed to survey respondents on its own – not on a pack of cigarette – which may have influenced how respondents viewed the label. However, optometrists are engaged daily in providing health advice, and their opinions on health communication including message content is very relevant to health authorities. Future studies should explore if this HWL did influence or impact patients’ decisions on seeking treatment for AMD. Further, it will be important to measure how this HWL is perceived by cigarette smokers and non-smokers. Further understanding how health effects associated with advanced age (such as AMD) influence youth or young adults would be important.

Optometrists are well positioned to address tobacco use with their patients and it is suggested that smoking cessation counseling by health professionals is effective in increasing cessation rates among smokers [20] and must be viewed as a standard of practice for every health professional [21]. Furthermore, the Health Canada HWL related to vision loss may help facilitate discussions with patients about tobacco use [22]. In Australia it has been shown that HWLs have increased smokers’ knowledge of smoking’s causal association with vision loss although the connection between smoking and vision loss remains unclear. Therefore, primary care providers like optometrists can play an important role in educating their patients about the causal association with cigarette smoking and the development of AMD.

## Conclusions

The findings from this study will help inform the development of other HWL in other jurisdictions. Over 120 countries have some form of HWL on their tobacco products and more than 60 now include pictorial HWL; however, very few jurisdictions include messages about blindness [23]. This number is anticipated to greatly increase since more than 170 countries have ratified the World Health Organization’s Framework Convention on Tobacco Control which obligates parties to introduce HWL on no less than 30 % of the principal display areas [24]. Research has shown that the effectiveness of HWLs tends to be universally accepted [25], therefore findings from this study has implications for the world-wide community. Future development of eye and vision related pictorial warning labels should include eye care practitioner input to ensure that important and accurate information is included. Similarly all HWL development should engage appropriate health experts.

## Abbreviations

AMD: Age-related macular degeneration
CCSRI: Canadian Cancer Society Research Initiative
HWL: Health warning label
ORE: Office of research ethics
